# How bone mis‐segmentation affects MRI‐based sCT dosimetry in head and neck radiotherapy: A CT‐referenced study

**DOI:** 10.1002/acm2.70722

**Published:** 2026-08-24

**Authors:** Laura Sayaque, Nils Tanneau, Charlène Bouyer, Benjamin Leporq, Oliver Hamelin, Frank Pilleul, Vincent Gregoire, Olivier Beuf

**Affiliations:** ^1^ INSA‐Lyon, Université Lyon 1 CNRS Inserm CREATIS UMR5220 U1294 Villeurbanne France; ^2^ Department of medical physics Centre de lutte contre le cancer Léon Bérard Lyon France; ^3^ Department of radiology Centre de lutte contre le cancer Léon Bérard Lyon France; ^4^ Department of radiotherapy Centre de lutte contre le cancer Léon Bérard Lyon France

**Keywords:** bone classification, dosimetry, head and neck radiotherapy, treatment planning

## Abstract

**Background:**

Magnetic resonance imaging (MRI) is commonly used for head‐and‐neck (H&N) delineation in radiotherapy (RT) owing to the lack of contrast in computed tomography (CT) images. MRI‐based dosimetry is not yet fully developed for the H&N area due to the inter‐patient variability and the difficulty of accurately quantifying cortical bone based on MRI measure.

**Purpose:**

Despite the difficulty of cortical bone segmentation with nearby air area, most papers in the literature show dosimetry results very close to the reference CT scan dosimetry. This study evaluated the impact of bone segmentation on dosimetry.

**Methods:**

A home‐made phantom was used to evaluate the Hounsfield unit (HU) values of different tissue at diverse scanner energies. Different dosimetry plans were considered and compared with the reference CT‐based RT plan used for the treatment: CT images with cortical bone voxels assigned to the density of water and three based on MR images using a density assignment (DA) method for synthetic CT (sCT) generation with various bone attribution. Images from 24 patients were compared using mean absolute error (MAE). The generated dosimetry parameters were compared on the basis of dose–volume histogram values and 3%/3 mm gamma pass rates.

**Results:**

Among the sCT, the best MAE were found for the DA sCT without bone. The mean of the median dose difference for the planning tumor volume (PTV) was less than 2% for all sCT. Mean gamma pass rate for the MR‐based sCTs were comparable.

**Conclusions:**

The DA of the bone can be of interest if correctly segmented, but in the absence of certainty, an overestimation will lead to significant errors while an underestimation gives results close to the reference. The relevance of very precise sCT can be questioned if it requires too many resources and overlooks patients with artifacts.

## 1 INTRODUCTION

For patients undergoing radiotherapy treatment, the workflow from structures delineation to dosimetry treatment planning is currently carried out on computed tomography (CT) images because their intensity values are directly linked to electron density—a prerequisite for dosimetry planning.

However, soft tissue contrast in CT images can lead to uncertainties in the delineating of the organs at risk (OAR) and of the tumor volume(s) (TV(s)),^1^ which may compromise treatment planning.^2^ In the head and neck area, for example, the numerous OAR are challenging for delineation but they are necessary for functions such as speech, mastication and respiration. Magnetic resonance imaging (MRI), offers superior soft‐tissue contrast, has been used in this area, but it induces additional difficulties.

Using MRI to delineate structures requires registration to the CT images used for dose planning, which can introduce significant errors,^2^ especially if the MRI is not acquired in the treatment position with immobilization devices. In these conditions, an MRI‐only workflow would require an MRI‐based dose planning, however the MRI images intensity is not directly correlated to electron density and therefore cannot be used for dose planning. Post‐treatment methods based on non‐CT images have been developed to generate synthetic CT (sCT), expressed in Hounsfield units (HU), mimicking CT contrast and values, thereby facilitating the treatment planning.^3^, ^4^, ^5^

Several methods exist for generating an sCT, including density assignment,^3^, ^6^, ^7^ atlas registration,^4^, ^8^ and deep learning.^8^, ^9^, ^10^ In recent years, deep learning methods have garnered significant interest within the community with many models being developed. However, most of them overlook patients with artifacts or peculiar anatomies to create homogeneous datasets, which limits the generalizability of both the models and their results to other datasets acquired with different parameters or using more advanced hardware.^11^ Given that most H&N cancer patients have dental artifacts, most datasets present inhomogeneities. Another problem related to this area is the heterogeneity of the tissue types. Cortical bone has a low proton density and a very short transverse relaxation time, which makes it difficult to measure with clinical MRI sequences and challenging to use for MRI‐based treatment planning. To measure the low proton‐density and short‐T_2_ MR signal from the bone, dedicated sequences are required, which are not always available on the clinical systems.

Current dose planning software requires an sCT where the intensity values of anatomical structures are expressed in HU, and are therefore related to electron density,,, but the National Bureau of Standards^12^ shows that for applications with high energy such as radiotherapy, the mass attenuation coefficient of bone is nearly identical to that of water. The importance of accurate bone segmentation can be questioned, since imaging bone with MRI is challenging. Thus, the impact of segmentation errors needs to be investigated.

This study evaluates the impact of including or excluding bone classes in both CT and sCT generated from MRI using a density assignment method.

## 2 MATERIALS AND METHODS

### 2.1 Patients and acquisition protocol

This study included a cohort of 24 patients with nasopharyngeal, oropharyngeal, laryngeal, or oral cavity squamous cell carcinoma undergoing radiotherapy treatment using a standardized imaging protocol at a single center. The study protocol was approved by the local ethics committee, and written informed consent was obtained from all patients before inclusion in the study.

The protocol included MRI acquisitions with clinical routine sequences for structure delineation (T_1_‐weighted turbo‐spin‐echo (TSE), T_2_‐weighted TSE, and gadolinium‐injected 3D T_1_‐weighted star‐VIBE (volumetric interpolated breath‐hold examination)) and more specific sequences for sCT generation (qDixon 3D VIBE and Ultrashort Time Echo (UTE) spiral VIBE^11^) on a 3T Siemens Magnetom Vida (Siemens Healthineers, Erlangen, Germany) system. All MR images were corrected for magnetic field inhomogeneities and gradient non‐linearity induced distortions using the built‐in 3D correction of the MRI scanner.^13^

A conventional CT scan (120 kV, 1‐mm slice thickness) with iodine contrast injection was performed on a Siemens Somatom Go Sim (Siemens Healthineers, Erlangen, Germany) and served as the reference for comparison with MRI‐based sCT for each patient. For patients with dental implants, an iterative Metal Artifact Reduction (iMAR) reconstruction was used.

To ensure reproducible positioning between imaging and treatment and to reduce the need for numerical co‐registration, a radiotherapy immobilization equipment was used for all the acquisitions. It is composed of a five‐point fixation immobilization mask, a fixation table, and a rigid radiotherapy table. As the usual diagnostic head and neck dedicated coil was not compatible with the immobilization mask, the 10‐channel lower part of a head coil (Head/Neck 20 shim MR Coil 3T), which includes shim coils and a 18‐channel flexible anterior body coil (Body 18 MR Coil 3T), were used. Small foam sponges were placed on the mask to elevate the body coil ensuring it did not contact the patient's nose. The acquisition setup was further described and illustrated in a previous manuscript.^7^In routine clinical practice, OAR delineation is automatically computed using TheraPanacea Annotate® (TheraPanacea, Paris, France) on CT images and manually corrected when necessary. The TVs are manually delineated on the CT images assisted by the T_1_‐weighted star VIBE gadolinium‐injected images as well as T_1_‐ and T_2_‐weighted TSE by radiation oncologists with over 15 years of experience, in accordance with international guidelines.^14^

### 2.2 Bone in CT and in MRI

The CT voxel intensities are expressed in HU. These values can be computed for each voxel of the image using Equation 1.

(1) $$HU=1000*\frac{\mu -\mu_{water}}{\mu_{water}}$$

where µ is the linear attenuation coefficient of the considered tissue expressed in m^−1^ and $\mu_{\mathrm{water}}$ is the linear attenuation coefficient of water. Because the linear attenuation coefficient depends on the atomic composition of the tissues, these values directly reflect tissue properties of radio‐transparency, which varies with the energy of the emitted photon beams.

The mass energy absorption coefficient µ_en_ expressed in m^2^/kg can be linked to the linear attenuation coefficient with Equation 2.

(2) $$\mu_{\mathrm{en}}=\frac{\mu }{\rho_{\mathrm{tissue}}}$$

where ρ_tissue_ is the density of the tissue. This coefficient does not consider the thickness of the tissue crossed by the beam.

The CT scan energies are typically below 0.20 MeV, and the radiotherapy treatment beams are usually up to 6 MeV. The National Bureau of Standards Handbook^12^ provides the mass energy absorption coefficient for the air, water, compact bone and muscle. At imaging energy, the µ_en_ values are 0.00231, 0.00252, 0.00383, and 0.00252 respectively. At treatment energy, they decrease to 0.00163, 0.00180, 0.00178, and 0.00178 respectively. The difference between water and bone mass energy absorption coefficients decreases substantially from low to high energies.

**FIGURE 1:**
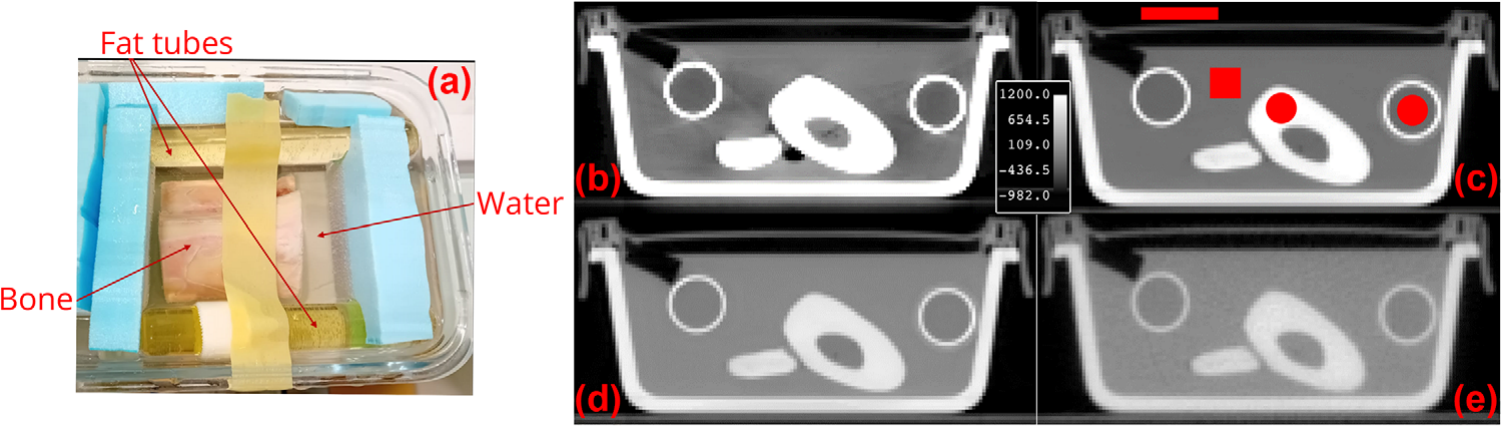
Photograph of the phantom designed for this study (a) |CT images of the phantom at 40 keV (b), 100 keV (c), 190 keV (d), and 1000 keV (e) |An arbitrary ROIs in red to compute the mean intensity for each class.

To validate these findings, a phantom was designed using tap water, two tubes of sunflower oil, and a marrow bone from a butcher. Dual‐energy CT was performed with reconstruction of the images at different energy levels from 45 to 190 keV with a spatial resolution of 1.1719 × 1.1719 × 3 mm^3^. The same phantom was scanned on a tomotherapy machine TomoTherapy® (Accuray, Sunnyvale, USA), which includes a high‐energy scanner with a spatial resolution of 0.7634 × 0.7634 × 2 mm^3^ and a polychromatic beam with a mean energy of 1 MeV.^15^ The phantom developed in this study is shown in Figure 1(a).

In MRI, the signal of cortical bone is not easy to measure. This is because the images intensity relies on hydrogen proton density, and bones contain very few hydrogen atoms with a very short transverse relaxation time (T_2_) component (< 1 ms), especially within cortical bone. To measure it, the echo time needs to be as close to zero seconds as possible. The UTE spiral VIBE sequence allows for very short echo times, as short as 30 µs, which enables to approach a measure of the cortical bone signal.

Considering the trabecular bone subclass, it is composed of both bone and marrow. It contains more fat than cortical bone and its signal can be measured with T_2_* mapping, which is reconstructed from a qDixon sequence.

### 2.3 Density assignment method

The density assignment method is used to evaluate the impact of inaccurate bone segmentation on the dosimetry. This method consists of defining several classes of tissues, segmenting them, and assigning them tissue‐specific HU values. To determine the value to be assigned to each class, we used the 3D Slicer software (https://www.slicer.org/), which includes a segmentation tool. We were able to determine HU ranges or means for the following tissue classes of air, water, fat, trabecular bone, and cortical bone. For air and cortical bone, a single value was assigned; for trabecular bone, water and fat, a range of values was assigned as follows:

Air: ‐1000 HU;
Water/Fat: From ‐100 to 100 HU;
Trabecular bone: From 200 to 900 HU;
Cortical bone: 1000 HU

Water and fat were jointly segmented using the qDixon sequence. This sequence reconstructs images of water and fat only, which can be combined to compute the proton density fat fraction (PDFF) following the formulas in Equation 3.

(3) $$PDFF=\frac{I_{fat}}{I_{fat}+I_{water}}$$

When the PDFF‐value is 0, the voxel contains only water whereas a PDFF‐value of 1 indicates only fat.

The transverse relaxation time value of trabecular bone at 3T is between 9 and 11 ms,^16^ providing a low and high threshold values that were applied on the T_2_* images of the qDixon sequence. The cortical bone signal was extracted using a threshold applied on the difference image derived from the Spiral VIBE sequence. The air signal was extracted using a threshold value of 55 to the UTE images. Dental artifacts were not treated as a separate class. Due to the difficulty in segmenting them, affected voxels were primarly assigned to cortical bone.

Since segmentation of the classes was performed using several sequences (qDixon fat, qDixon water, qDixon T_2_*, and UTE), some voxels may have been assigned to multiple classes. In that case, as a simplifying assumption, the segmentation values were summed to obtain the final sCT. The two sequences used in the MRI protocol were acquired with the same geometrical parameters, eliminating the need for co‐registration was needed.

To evaluate the impact of bone, three types of sCT were generated from MR images along with a CT‐based reference. The CT‐based sCT referred to as “CT_no bone_” used the reference CT modified using HU thresholding with a threshold value for cortical bone set to 700 HU, selected voxel values were set to water density. For patients with dental artifacts, the reference CT used was iMAR corrected. For each of the 24 patients, the generation of these images was performed with Matlab (MathWorks, Natick, MA, USA) algorithms.

The first type of sCT contains only the classes of air, water, and fat, called “DA_no bone_”, segmented from the qDixon sequence images.

The second type of sCT contains the typical classes of air, water, fat, and trabecular and cortical bone, called “DA”, segmented on the qDixon and the UTE sequence images.

The last type of sCT contains the same classes, with a wider MRI threshold for the trabecular bone, from 9–11 ms to 8–12 ms, called “DA_more bone_”, which has an impact on the trabecular bone class. The percentage of voxels added to the bone classes was assessed in each patient.

All reconstructed images were registered and compared voxel‐wise with the original treatment CT using mean absolute error (MAE) computation.

### 2.4 Dose planning

OARs and TVs were delineated on the CT images and propagated to the sCT images. Treatment plans were generated for volumetric modulated arc therapy (VMAT) under a set of dose constraints on the median dose for the TVs and the mean and/or near maximum‐dose for the OAR, particularly for organs near the target, defined by the radiation oncologist. Dose planning was performed using MONACO (Elekta, Stockholm, Sweden), a Monte Carlo‐based clinical treatment planning system while accounting for system constraints. Dose computation and optimization were performed on the CT images. The same treatment plans were then recomputed without re‐optimization on the MRI‐based sCT and CT_no bone_ images. Algorithm parameters are summarized in Table 1.

**TABLE 1:** Monte carlo algorithm parameters used for dose calculation.

Grid spacing (cm)	0.3
Statistical uncertainty (%) per calculation	1
**Sequencing parameters**	
Number of arcs	2 to 4
Max number of control points (per arc)	150 to 180
Min segment width (cm)	0.5
Fluence smoothing	low

The dose—volume histograms (DVH) were computed and compared for each dose planning. We evaluated the differences in dose for 2% of the volume (D_2%_) for serial OAR, the so‐called near‐maximum dose: mandible, brainstem, esophagus, thyroid gland, larynx, lips, spinal cord, and trachea. The mean dose (D_mean_) was computed for parallel OAR: esophagus, oral cavity, left and right parotids, left and right submandibular glands. Finally, we assessed the differences in dose for 50% of the volume (D_med_) of the following TVs: gross tumor volume (GTV), high‐risk planning tumor volume of 70 Gy (PTV_HR_), low‐risk planning tumor volume of 54.25 Gy (PTV_LR_) and clinical target volume (CTV). Dose differences were considered acceptable when inferior to 2%, following the criterion identified by the ESTRO 2023 survey on the use of sCT for MRI‐only radiotherapy.^14^ Lastly, we computed the dose at 95% of the PTV, this value needs to be superior to 95% of the prescribed dose to be accepted in a clinical context. Gamma pass rates were computed for each patient with a 3%/3 mm criterion using the reference module pymedphys 0.38.0^17^ on Python. Wilcoxon tests were computed to compare all metrics between the methods using the python module Scipy 1.17.

## 3 RESULTS

### 3.1 Patients and acquisition protocol

Four women and 20 men took part in the study, with a mean age of 66.4 years; 13 patients had dental implants, which led to more or less important artifacts that were qualitatively assessed in both CT and MRI. For CT images, the iMar corrected images were used while in MRI no specific correction was implemented. This choice allowed to evaluate the method on patients affected by artifacts to various degrees and investigate its capability in an anatomical area often impacted by artifacts caused by metallic implants.

### 3.2 Bone signal intensity in CT and in MRI

Figure 1 shows CT slices of the phantom for different energy values (b, c, d) and for tomotherapy imaging beam (e).

**FIGURE 2:**
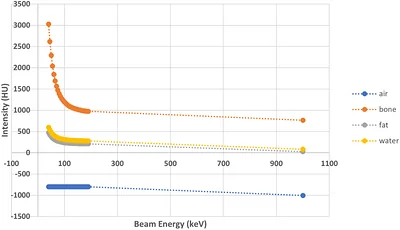
HU intensities for different energy values and for each phantom class.

A mean region of interest (ROI) of approximately 100 voxels (in red in Figure 1(c)) was drawn on three slices for each energy scan and each class of tissue. Position of the ROI was decided to avoid partial volume effects between tissues. Figure 2 shows the curves expressing the HU according to the photon beam energy for each phantom class ROIs.

Depending on beam energy, the voxel intensities of the bone class vary from 3028 to 763 HU, the water class voxels vary from 598 to 83 HU, the fat class voxels vary from 473 to 27 HU, and the air class voxels vary from −799 to −1007 HU. Normalizing the HU values of the tissues with the HU value of the bone, as shown in Equation 4, highlights the decrease of the difference between bone and water with increasing energies. Table 2 shows the mean normalized intensity values for tested energies.

(4) $$\begin{array}{cc} & Hi,k_{normalized}=\frac{H_{i,k}}{H_{bone,k}}\\ & \;\mathrm{with}\;i\;\mathrm{the}\;\mathrm{tissue}\;\mathrm{and}\;k\;\mathrm{the}\;\mathrm{beam}\;\mathrm{energy} \end{array}$$

**TABLE 2:** Mean normalized intensity values.

Beam energy (keV)	Air	Bone	Fat	Water	Water (theoretical)
40	0	1	0.33	0.36	—
100	0	1	0.52	0.56	0,46
190	0	1	0.57	0.61	—
1000	0	1	0.58	0.62	0,72

The theoretical normalized HU values for water at 100 and 1000 keV were computed using the mass energy absorption coefficients from the National Bureau of Standards handbook^12^ and the density of air (1.29 kg/m^3^), compact bone (1500 kg/m^3^) and water (997 kg/m^3^).

### 3.3 Density assignment method

**FIGURE 3:**
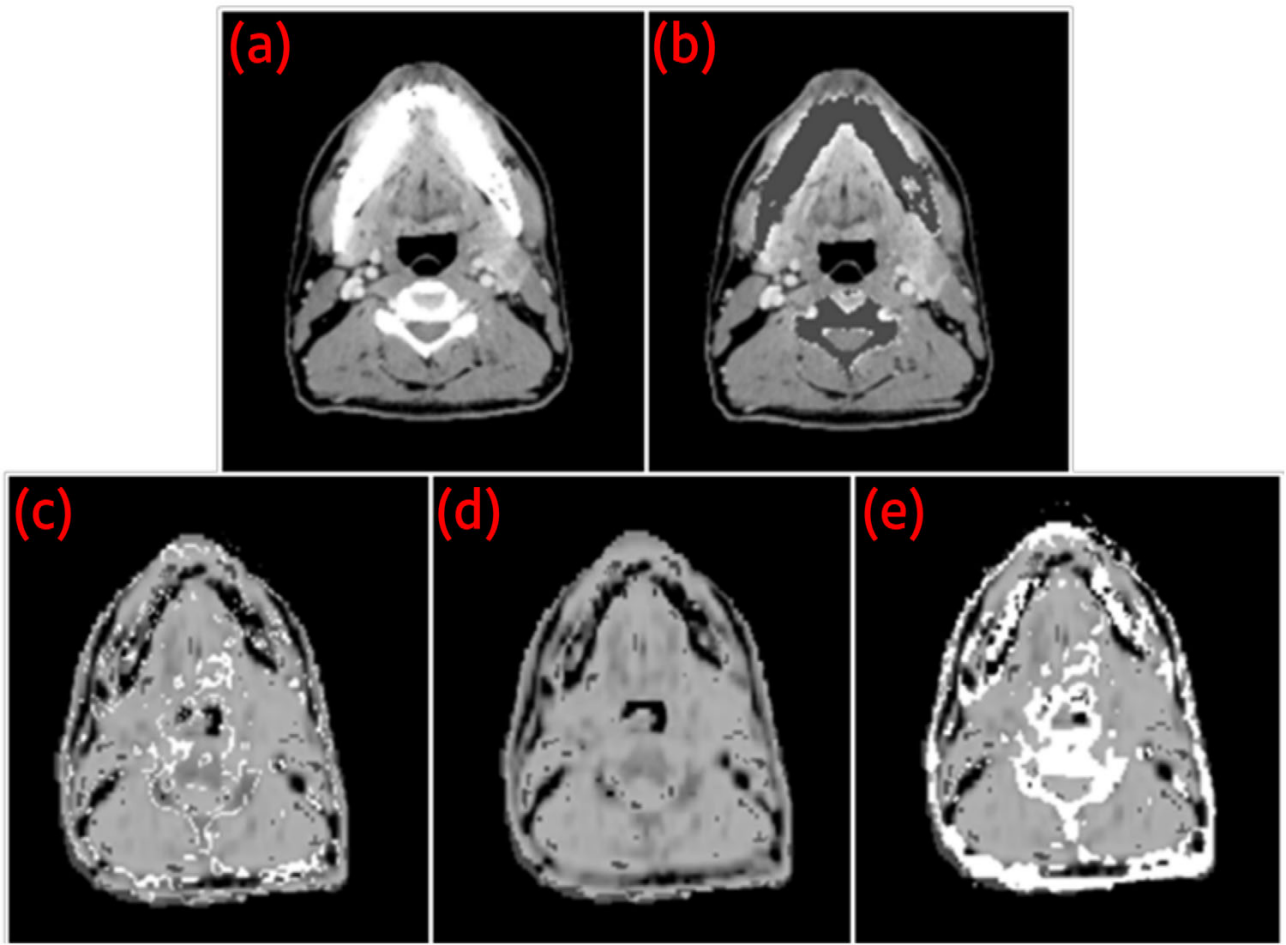
Slice of the volume of the same patient for CT (a), CT without bone (b), DA sCT (c), DA sCT without bone pixels (d), and DA sCT with bone overestimated (e).

Four series of sCT were generated for the 24 patients. Figure 3 shows an axial slice for each sCT generated with the original CT.

The images in Figure 3(b) and (d) are similar, while 3(c) shows a bone class with very few high voxel values inside the bone. Figure 3(e) shows very high intensities for the voxels at the interface with air around the patient. The proportion of voxels assigned to bone on DA_more bone_ was compared to the proportion in DA, resulting in a mean increase of 5.8 ± 3.3% bone‐assigned voxels in DA_more bone_. This increased proportion was mostly composed of voxels located at air‐bone interfaces. This proportion was also computed for DA_no bone_ and compared with DA, resulting in 1.7 ± 0.6% more voxels assigned to bone in the later. The interval of HU values found in each sCT was checked to verify that no voxels were assigned to unphysical HU values (outside of the [‐1000;2000] HU interval).

MAE computation with the treatment CT resulted in mean MAE values of 270 ± 59 HU for DA, 197 ± 45 HU for DA_no bone_, 386 ± 82 HU for DA_more bone_, and 66 ± 10 HU for CT_no bone_.

The mean MAE is very high for DA_more bone_, while it is the lowest for CT_no bone_. The MAE value in CT_no bone_ is 66 HU suggesting that bone volumes have low impact on HU differences in the patient's overall volume. This idea can also be observed when comparing MAE values between DA and DA_no bone_ where the difference is 73 HU.

### 3.4 Dose planning validation

**FIGURE 4:**
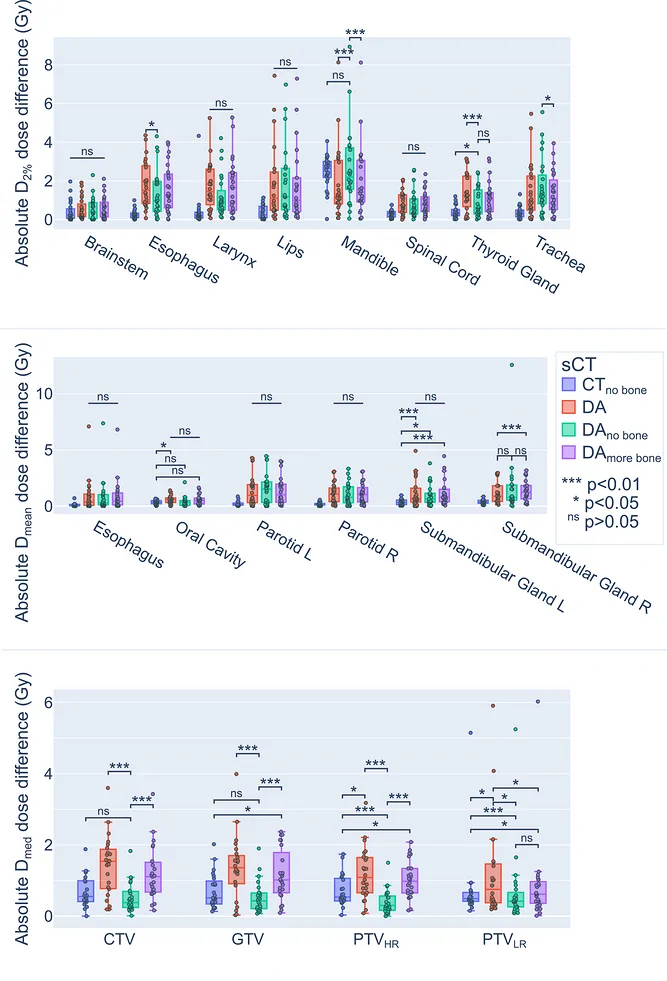
Absolute dose differences (in Gy) in OAR and tumoral volumes for the various sCT methods studied, points represent the sample distributions.

The dosimetry plans for the 24 patients were computed, the dose differences presented in Table 3 were extracted from the DVH for the OAR and TVs. These dose differences are presented in %, as a percent of the treatment prescribed dose (70 Gy most of the time). Figure 4 shows the absolute dose differences in Gy for the studied OAR and TVs, results of statistical tests have been also reported. Outliers seen on the lips, mandible and esophagus boxplots in Figure 4 correspond to the same patient that was characterized with moderate dental artifacts in both MR and CT. Other outliers in the other OAR and TVs boxplots do not belong to patients that were identified with important dental artifacts.

**TABLE 3:** Dose differences at 2% of the volume, at the mean dose for OAR noted on the first column and at 50% of the volume for TVs in percent.

Tissue	DA	DA_no bone_	DA_more bone_	CT_no bone_
**D_2%_**				
Brainstem	0.80	0.76	0.83	0.57
Esophagus	2.44	1.77	2.13	0.39
Larynx	2.45	1.82	2.60	0.56
Lips	2.69	2.73	2.57	0.56
Mandible	2.81	3.93	2.94	3.55
Spinal cord	1.18	1.10	1.21	0.43
Thyroid gland	1.81	1.20	1.47	0.59
Trachea	1.95	2.37	1.91	0.53
**D_mean_**				
Esophagus	0.03	0.20	0.12	0.02
Oral cavity	0.36	0.23	0.38	0.30
Parotid L	1.15	1.10	0.74	0.16
Parotid R	0.10	0.35	0.00	0.15
Submand gland L	2.38	0.07	0.67	0.36
Submand gland R	0.59	0.62	0.83	0.34
**D_med_**				
CTV	2.07	0.76	1.73	0.92
GTV	1.96	0.73	1.69	0.94
PTV_LR_	1.28	0.70	0.84	0.71
PTV_HR_	1.68	0.52	1.46	0.96

The mean dose differences between CT and sCT for the 24 patients are very low for most of the OAR and TVs. The two boneless sCTs (CT_no bone_ and DA_no bone_) show dose differences inferior to 1% for every TV.

**FIGURE 5:**
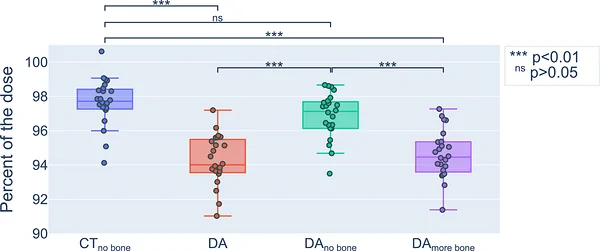
Percent of the dose for 95% of the PTVHR for the four types of sCT‐based dosimetry, points represent the sample distribution.

Figure 5 shows the dose at 95% of the PTV_HR_. For DA, 38% of the patients have dose superior to 95% of the prescribed dose, for DA_no bone_ 92% of the patients have dose superior to 95% of the prescribed dose, for DA_more bone_ 42% of the patients have dose superior to 95% of the prescribed dose and for CT_no bone_ 96% of the patients have dose superior to 95% of the prescribed dose. Wilcoxon tests resulted in a significant difference between the dose at 95% of the PTV_HR_ of CT_no bone_ and the dose of the other tested methods (*p* < 0.01) with the exception of DA_no bone_ (*p* > 0.05). Significant difference between DA_no bone_ and the other MRI‐based sCT was also obtained (*p* < 0.01). Results of the Wilcoxon tests are reported in Figure 5.

**FIGURE 6:**
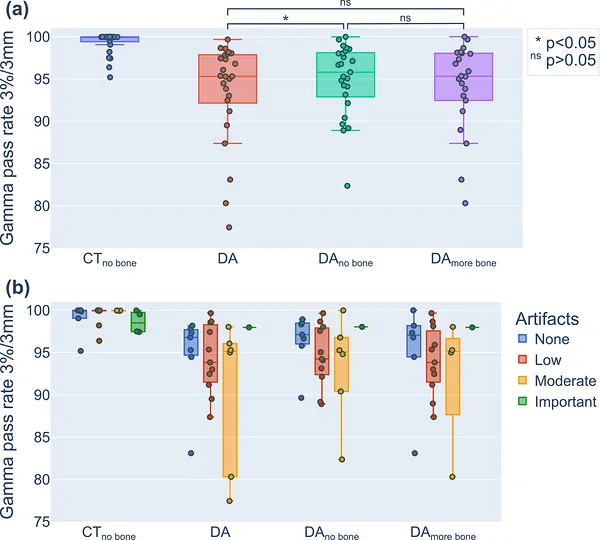
3%/3 mm gamma pass rates values for the tested sCT dosimetry with statistical tests results (a)| Gamma pass rate values with distinction between qualitatively assessed artifacts (b).

The gamma pass rate results are showed in Figure 6(a) shows the gamma pass rate values for the different tested sCT while Figure 6(b) shows the distribution of values depending on the qualitatively assessed MR artifacts for the DA‐based sCTs and the CT artifacts for CT_no bone_ DA gave a mean value of 93.5 ± 6%, DA_no bone_ a mean value of 94.9 ± 4.3%, DA_more bone_ a mean value of 94.3 ± 4.7% and CT_no bone_ a mean value of 99.3 ± 1.3%. Wilcoxon tests resulted in a significant difference between the gamma pass rates of CT_no bone_ and the other tested methods (*p* < 0.01) and in a significant difference between DA and DA_no bone_ (*p* < 0.05). Other results of the Wilcoxon tests are reported in Figure 6(a). Artifact's repartition on Figure 6(b) suggests an increase of the deviation in gamma pass rate values for patients with increasing artifact importance in all tested MR‐based sCT although only one patient was identified with important MRI artifacts.

## 4 DISCUSSION

In the radiotherapy workflow, the use of MRI for dose planning is becoming more prevalent and some methods have even been implemented in commercial systems for clinical use.^8^, ^18^ However, extending this approach to the head and neck region is not straightforward due to the high number of OARs, the complex anatomy, and the numerous tissue types; thus, clinical implementation remains a long process for all developed techniques. To the team's knowledge, only one software package capable of processing head and neck MR images, regardless of the MRI scanner manufacturer, is commercially available.^19^ Moreover, cortical bone signal is difficult to measure using MRI and methods such as density assignment require specific sequences solely for this purpose. Deep learning based methods also often require the implementation of dedicated networks for that matter.^20^ The treatment planning systems are designed for CT images, which convert HU values to electron density using calibration curves. With technologies such as MRI‐Linac, an MRI‐only photon radiotherapy workflow could become feasible, without the need for a sCT for treatment repositioning. Although the contrast between bone HU values and soft tissue is very high in low‐energy CT, this contrast decreases at higher energies, such as those used in radiotherapy treatment, raising questions of the impact of accurate bone assignment in sCT. Our study phantom study demonstrated that the contrast between bone and soft tissues decreases significantly between low energies (< 100 keV) and high energies (> 1 MeV) and remains relatively similar between 100 keV and 1 MeV. The results obtained differ from the data computed with National Bureau of Standards^12^ values because the tomotherapy system has a polychromatic beam with an estimated energy of 1 MeV; this value may have been over‐estimated, especially since bone phantom is inert animal bone. Also, the tissue mass density affects HU contrast of CT images while mass energy absorption coefficient (provided by the National Bureau of Standards^12^) does not account for it.

Current treatment planning systems require CT or sCT images in HU to perform dosimetry. The results of our density assignment method must to be compared with literature data, however, the head and neck area is a complex anatomical location, and methods relying on databases and generalized models are difficult to adapt to the wide variety of cancers in this area. For instance, atlas‐based registration methods are rarely used for this location. The few existing articles on this method^8^, ^21^only display gamma index maps, without providing gamma pass rate values, which makes comparisons with other methods difficult. Deep learning‐based methods have flourished over the past five years^9^, ^10^, ^19^, ^20^, ^22^, ^23^, ^24^, ^25^ producing sCT images that closely resemble CT scans using increasingly complex models. The clinical translation of these methods appears limited by the need for a large datasets and poor adaptability for atypical anatomies, artifacts and variations across centers or systems. While the same neural network could theoretically be used across different centers, it would require trained with center‐specific datasets and may still fail to adapted to certain patients. Moreover, these methods often exclude patients with significant dental artifacts, which are common in this region as shown in our study where 54% of the cohort exhibited such artifacts.

Notable MAE results have been reported for atlas‐based and deep learning‐based methods,^8^, ^26^, ^27^, ^28^ e.g., 116 HU,^4^ 113 HU,^29^ or 108 HU^21^ for the former and between 65 HU^30^ and 131 HU^31^ for the latter. A study using a commercially available solution reported a median MAE value of 93HU^19^ This metric depends heavily on the registration between the compared volumes, and the quality of bone assignment also plays a role, as demonstrated with our density assignment method: the DA_more bone_ yielded a mean MAE of 386 ± 82 HU, whereas the DA_no bone_ obtained a mean MAE of 197 ± 45 HU. The MAE of the CT_no bone_ method of 66 ± 10 HU was obtained without the uncertainty of co‐registration with the CT, further demonstrating how bone differences can relatively impact on this metric. This value is comparable to the MAE of the best deep learning‐based methods, suggesting that bone absence may not impact MAE more than MRI‐based methods do.

The differences between CT and sCT are clear during the voxel‐wise comparison, but they tend to decrease in the following step of dosimetry; the MAE is based solely on sCT, and thus a higher MAE does not necessarily result in a higher dose difference and vice versa. The most accurate and relevant indicator for comparing the dosimetry of the four methods is the dose difference analysis. The values are directly extracted from the dose–volume histogram, which leads to fewer errors than metrics such as the MAE or the gamma pass rate that depend on a registration between the different volumes to be compared. Most of the recent deep learning‐based articles are limited to MAE comparison, the dosimetry is not computed and compared with the one used for treatment; thus, the quality of the method is only based on the resemblance between the CT and the sCT.^10^, ^22^, ^23^, ^26^, ^32^

The dose difference for the high‐risk PTV ranges from 0.5% to 1.6% for the four tested sCT —‐ and for the two boneless methods (DA_no bone_ and CT_no bone_) they are below 1%. These results differ from the literature for head and neck radiotherapy, since the articles in the literature on bulk density assignment only compare sCT with water equivalent (WE) density and one or several sCT with different bone density assigned.^3^, ^6^, ^22^ Our DA_no bone_ sCT distinguishes water from fat, leading to slightly different results than a WE density assignment. Karotki et al.^3^ report a 4–5% dose difference with their WE method, and Chin et al.^6^ reported a 28% coverage difference for a PTV of 70 Gy (PTV_70_). These results need to be discussed because the algorithms used for the computation of the dose are less precise than the Monte Carlo algorithm used at the treatment center.^33^ When referring to the dose difference acceptance criteria defined in the Estro 2023 survey,^14^ none of the tested sCT would be acceptable as some dose difference are superior to 2%. Especially in the mandible for the DA_no bone_ and CT_no bone_. Among the MR‐based sCT, DA_no bone_ would be the closest for clinical acceptability with three OAR dose differences going over the 2% threshold and none in TV.

As expected, the DA_more bone_ gave the worst results in almost all studied metrics. These results could have been partly influenced by the voxels classified as bone at the air‐skin interface. Such segmentation errors arise because bone signal is close to air signal in MRI and a wrongly set automatic segmentation tool would count them as bone. The sum‐on‐overlap approximation could have impacted the results, especially in DA_more bone_ as voxels at interfaces could have been assigned unfit values. This is suggested by the increased dose difference in the esophagus but not significant difference was observed with the one computed for DA. Implementing a priority rule in tissue masks might help avoid such issue and address the limitations induced by the sum‐on‐overlap method. The classic DA method gave higher dose differences than the CT for all the TVs and for several OAR. Most of the literature on head and neck radiotherapy, has results are similar to ours. For instance, with a generative adversarial neural network (GAN), Klages et al.^34^ obtained a dose difference of less than 2% for OAR and less than 0.8% for PTV_70_ (equivalent to the PTV volume in this study).

The results for the dose at 95% of the PTV_HR_ also reflect the negative impact of an overestimation of the bone in DA_more bone_, with only 40% of the patients passing the criterion of 95% of the prescribed dose. The only patient below the threshold of 95% for the CT_no bone_ dosimetry had severe artifacts which impacted the delineation and was at 94.1% of the prescribed dose (70 Gy). The two patients under the 95% bar for the DA_no bone_ were also impacted by artifacts and at 93.5 and 94.7% of the prescribed dose.

The gamma pass rate values obtained in this study are mostly under the ones found in the literature. For a 3%/3 mm criterion, Thummerer et al.^30^ obtained 97% and Dinkla et al.^5^ obtained 98.7% with deep learning methods, but some articles show close results, as Deng et al.^24^ with at least 95%. Our density assignment methods are close to 95%, especially the DA_no bone_. The CT_no bone_ reached the same values as the best paper in the literature: Peng et al.^27^ with 99.6% or Guerreiro et al.^4^ with 99%. The impact of artifacts on dosimetry can be discussed from the gamma pass rates values. For patients with moderate to severe important artifacts, values were less robust than the ones from patients with none to low artifacts although the small number of patients with important severe MR artifacts in the cohort limits the conclusions that can be drawn from this analysis.

Finally, this study shows that even with large voxelwise differences between sCT and CT, as seen with the MAE, the dosimetry results are mostly consistent. The dosimetry on CT_no bone_ shows that removing only the bone leads to small dosimetric differences when compared with the CT‐based dosimetry. In MR‐based density assignment methods, removing the bone results in small but significant differences in dosimetry, suggesting an improvement compared to the classic approach. However, there is still a gap to clinical relevance as the dosimetric results are still below community‐defined thresholds for safe translation of sCT.^14^ To continue on this path of developing an MRI‐only workflow, the impact of MRI‐based modifications of the contours of the TV and the OAR should also be studied. This work is ongoing in our department.

## 5 CONCLUSION

In conclusion, this study demonstrates that imperfect bone delineation and HU assignment can significantly impact the generated sCT, whereas dosimetry is not equally impacted. Although bone HU values in low‐energy CT are very high, the contrast with soft tissues decreases at radiotherapy treatment energy levels. This raises the question of whether an extremely precise MR‐based sCT is necessary for head and neck photon radiotherapy treatment planning, given that it requires significant resources and multiple MRI sequences, when a slightly less precise sCT could yield dosimetry results comparable to those from CT.

## AUTHOR CONTRIBUTIONS

**Laura Sayaque**: Methodology; investigation; analysis; writing–original draft. **Nils Tanneau**: Methodology; analysis; writing–original draft; writing–revised draft. **Charlène Bouyer**: Methodology; investigation; writing–original draft. **Benjamin Leporq**: Methodology; writing–original draft; resources; supervision. **Olivier Hamelin**: Data acquisition; writing–original draft. **Frank Pilleul**: Methodology; writing–original draft. **Vincent Gregoire**: Methodology; writing–original draft; supervision. **Olivier Beuf**: Methodology; funding acquisition; writing–original draft; supervision.

## CONFLICT OF INTEREST STATEMENT

The authors declare no conflicts of interest.

## ETHICAL APPROVAL

The study was approved by the national ethics committee R201‐004‐314 and all patients provided their written informed consent

## Data Availability

The data that support the findings of this study are available from the corresponding author upon reasonable request. The data are not publicly available due to privacy or ethical restrictions.
